# What happens to your hearing if you are born blind?

**DOI:** 10.1093/brain/awt346

**Published:** 2014-01-13

**Authors:** Andrew J. King

**Affiliations:** Department of Physiology, Anatomy and Genetics, University of Oxford, Oxford, UK E-mail: andrew.king@dpag.ox.ac.uk

Our different senses often work together to enhance perception, for instance helping us to understand what was said by the person we are trying to talk to in a noisy bar, or to work out where that other voice came from. Indeed, cross-sensory interactions are so pervasive that many brain regions, including early sensory cortical areas that were previously thought only to process modality-specific information, are now known to receive multisensory inputs (Alais *et al.*, 2010). Another manifestation of the close relationship between the senses is seen in the sometimes profound changes in the way in which early blind or deaf individuals use their remaining senses. In this issue of *Brain*, Monica Gori and colleagues describe an unexpected example of such cross-modal plasticity by showing that congenitally blind subjects are severely impaired in their ability to perform an auditory spatial task.

Although studies of cross-modal plasticity in blind people have measured the performance of subjects in various auditory or tactile discrimination tasks, a lot of research in this area has focused on whether auditory spatial abilities change after loss of vision. One reason for this is that spatially-aligned visual cues can improve the accuracy of auditory localization (Shelton and Searle, 1980), whereas misaligned visual cues can bias or capture the perceived location of a sound source, as in the ventriloquist illusion (Bertelson and Radeau, 1981). Given the ample evidence for interactions between these senses, with vision tending to play the dominant role in resolving spatial conflicts between them and in aligning neural maps of space in the midbrain during development (King, 2009), it might well be expected that early loss of vision would result in impaired spatial hearing.

In fact, several previous studies (Röder *et al.*, 1999; Voss *et al.*, 2004) have reported the opposite result, with blind subjects performing as well as, or even better than, sighted controls when tested for their ability to localize sounds in the horizontal plane. Moreover, there is growing evidence that blindness (or deafness) can result in functional changes within the brain that are likely to underlie perceptual improvements in the intact sensory modalities. For example, improved auditory spatial abilities in blind people have been linked to the recruitment of occipital cortical areas deprived of their normal visual inputs (Collignon *et al.*, 2009), whereas studies in animals have shown that sound processing by neurons in auditory cortical areas can be enhanced in the absence of vision (Korte and Rauschecker, 1993; Petrus *et al.*, 2014).

In view of this, the finding by Gori *et al.* (2014) that an aspect of auditory spatial processing is impaired in congenitally blind people comes as something of a surprise. This is not obviously related to the age at onset or severity of blindness, as improved sound localization accuracy has been reported in both early and late blind subjects (Voss *et al.*, 2004) and even after blindfolding normal-sighted adults for just 90 min (Lewald, 2007). Instead, the fundamental difference between the findings from these studies seems to be related to the nature of the behavioural task that the subjects carried out.

In the earlier studies in which auditory localization performance in blind individuals was found to be as good as or better than normal, subjects were asked to turn toward the perceived location of the sound source from among an array of loudspeakers or to indicate whether the second of two consecutive sounds came from the same location as the first sound or a different one. In contrast, Gori and colleagues employed a more complex spatial bisection task that required their subjects to judge the relative position of the second sound source in a sequence of three sounds presented from different angles in the horizontal plane. They found that their congenitally blind patients either had significantly elevated thresholds relative to the normal-sighted control group or were unable to do the task at all. This in itself is remarkable as the differences reported in previous studies have tended to be more subtle.

The deficits observed by Gori and colleagues were specific to the spatial bisection task—which required a comparison of the perceived difference in location between the first two and last two sounds—as no differences were found between the blind and sighted groups when they switched to more conventional methods in which subjects were asked to point to the perceived source of a single sound or to carry out a minimum audible angle task. Importantly, the blind subjects were also unimpaired in their ability to perform a temporal bisection task—in which they had to indicate whether the middle of three sounds was closer in time to the first or last sound—suggesting that the spatial deficit was unlikely to reflect either a difficulty in understanding the task or in holding the sequence of sounds in working memory.

While the results of this study are clearly at odds with the commonly held view that blind individuals compensate for a lack of vision by developing enhanced non-visual abilities, supra-normal sound localization accuracy has usually been reported only for peripheral rather than central regions of space (King and Parsons, 1999; Röder *et al.*, 1999; Voss *et al.*, 2004). Because Gori *et al.* (2014) restricted their testing to the frontal ±25°, the possibility that they might have obtained a different result had they extended the range of their loudspeaker array cannot be ruled out. On the other hand, blind people tend to make less accurate elevation responses than sighted subjects when tested on or close to the midsagittal plane (Zwiers *et al.*, 2001; Lewald, 2002). Thus, it would appear that although blindness can lead to the emergence of superior auditory localization abilities at more peripheral locations, where visual cues are normally less readily available, loss of vision in the frontal region of space may have a detrimental effect on the development of accurate spatial hearing.

Gori *et al.* (2014) claim that their results support a role for vision during development in guiding the construction of a neural representation of auditory space. In particular, they argue that these findings are consistent with studies in animals demonstrating a dominant role for visual experience in merging maps of different sensory modalities in the superior colliculus. Establishing and maintaining the registration of these maps is challenging because spatial information is derived and represented by each sensory system in different ways. Thus, the locations of visual (and somatosensory) stimuli are encoded directly by the distribution of neural activity across the receptor cells and at successive stages of central processing, whereas sound-source location has to be computed within the brain by tuning neurons to particular combinations of spatial cues—such as the difference in sound level or timing between the ears—that result from the way sounds interact with the head and external ears. Aligning the maps in the superior colliculus therefore involves matching the sensitivity of neurons to auditory localization cue values with positions on the retina that correspond to the same region of space. Moreover, this relationship changes as the body grows.

Vision clearly plays an important guiding role in auditory map development, with early visual deprivation degrading the topographic organization of the auditory receptive fields of superior colliculus neurons to varying degrees (reviewed in King, 2009; Gutfreund and King, 2012). It is hard to argue on the basis of these studies, however, that vision is required for the construction of a map of auditory space in the brain. Instead, the availability of concurrent and generally more accurate visual spatial cues most likely helps to overcome the uncertainty and variability in the relationship between auditory localization cue values and directions in space. As a result, the developing auditory map is shaped so that it matches the visual field representation in the superior colliculus, thereby facilitating the integration of signals provided by the eyes and the ears about a common stimulus source (Gutfreund and King, 2012).

It is unclear that the physiological data from visually deprived animals would predict a profound impairment by congenitally blind subjects in spatial bisection, particularly as the accuracy of pointing toward a single sound source—a task arguably more closely allied to the sensorimotor orienting functions of the superior colliculus—was preserved. Nevertheless, these findings raise intriguing, and testable, questions about the nature of spatial representations in the brain and how they are refined by sensory experience. Moreover, if, as Gori and colleagues suggest, the impaired performance on the spatial bisection task in blind subjects reflects a lack of visual calibration of the developing auditory system, these deficits would presumably not be seen in individuals with late-onset acquired blindness.

Finally, the findings of this study have potentially important implications for rehabilitation strategies for visually impaired individuals. Information transfer between sensory modalities may allow other cues to partially substitute for vision in blind people, particularly if parts of the visual cortex have become colonized by auditory or tactile inputs. However, the use of ‘sensory substitution devices’ to provide blind people with increased spatial awareness via their hearing relies on the principle that the information they provide is reliable and accurate. The findings of Gori and colleagues question whether this is necessarily the case.

## Funding

A.J.K. is a Wellcome Trust Principal Research Fellow (WT076508AIA).
